# ELKS active zone proteins as multitasking scaffolds for secretion

**DOI:** 10.1098/rsob.170258

**Published:** 2018-02-28

**Authors:** Richard G. Held, Pascal S. Kaeser

**Affiliations:** Department of Neurobiology, Harvard Medical School, Boston, MA 02115, USA

**Keywords:** secretion, cellular traffic, synapse, active zone, ELKS

## Abstract

Synaptic vesicle exocytosis relies on the tethering of release ready vesicles close to voltage-gated Ca^2+^ channels and specific lipids at the future site of fusion. This enables rapid and efficient neurotransmitter secretion during presynaptic depolarization by an action potential. Extensive research has revealed that this tethering is mediated by an active zone, a protein dense structure that is attached to the presynaptic plasma membrane and opposed to postsynaptic receptors. Although roles of individual active zone proteins in exocytosis are in part understood, the molecular mechanisms that hold the protein scaffold at the active zone together and link it to the presynaptic plasma membrane have remained unknown. This is largely due to redundancy within and across scaffolding protein families at the active zone. Recent studies, however, have uncovered that ELKS proteins, also called ERC, Rab6IP2 or CAST, act as active zone scaffolds redundant with RIMs. This redundancy has led to diverse synaptic phenotypes in studies of ELKS knockout mice, perhaps because different synapses rely to a variable extent on scaffolding redundancy. In this review, we first evaluate the need for presynaptic scaffolding, and we then discuss how the diverse synaptic and non-synaptic functional roles of ELKS support the hypothesis that ELKS provides molecular scaffolding for organizing vesicle traffic at the presynaptic active zone and in other cellular compartments.

## 1. Introduction

Information transfer between neurons is largely carried out through synaptic transmission [1]. When a neuron is sufficiently depolarized, it fires an action potential that propagates down the axon into the presynaptic nerve terminals. In response to the depolarization induced by the arriving action potential, voltage-gated Ca^2+^ channels open, allowing influx of Ca^2+^ into the terminal. Synaptic vesicles then fuse in response to Ca^2+^ entry [2–5]. Notably, only a subset of synaptic vesicles, known as the readily releasable pool (RRP) of vesicles, is in a fusion competent, ‘primed’ state [6], and their fusion is triggered by Ca^2+^ binding to the vesicular Ca^2+^ sensor synaptotagmin [7]. Membrane fusion is mediated by protein interactions between vesicular and target membrane SNARE proteins that rapidly zipper to fuse the two membranes [2,8–10], a process that is guided by the S/M protein Munc18 [11,12]. Upon fusion pore opening, the vesicles release their neurotransmitter content into the synaptic cleft, and the neurotransmitter diffuses to bind to receptors on the postsynaptic cell.

Hallmarks of synaptic transmission are its speed and its spatial precision. The fusion process at a synapse only consumes hundreds of microseconds [13,14], and vesicles only fuse with the target membrane that is precisely opposed to the postsynaptic receptors [15,16]. However, SNARE proteins, SM proteins and synaptotagmin mediate fusion in many secretory cells where fusion is less spatially restricted and is executed on much slower time scales [17–21]. Additional protein machinery is, therefore, required to account for the speed and spatial precision of synaptic signalling. The presynaptic active zone fills this role, enabling the speed and precision of synaptic transmission [22,23]. We here define the active zone as the protein complex that is attached to the presynaptic plasma membrane opposed to the postsynaptic density. A key function of this complex is molecular scaffolding: the capture, anchoring and spatial organization of the components which execute the fusion of synaptic vesicles. In this review, we discuss recent studies of protein scaffolding within the active zone, focusing on the active zone protein ELKS to illustrate the significance and the diverse roles of presynaptic scaffolding. We further assess how redundancy in scaffolding across several protein families complicates the study of this hallmark feature of the synapse.

## 2. Active zone scaffolding is important for neurotransmitter secretion

**Figure 1.**
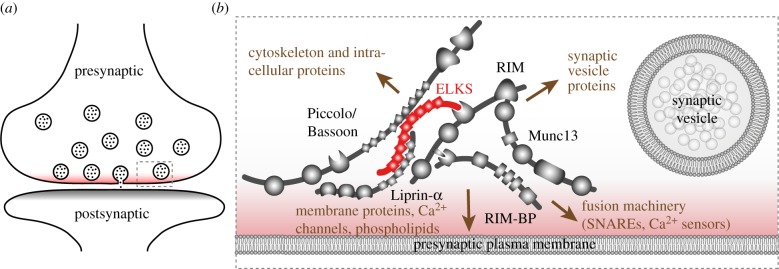
The presynaptic active zone. (*a*) Schematic of a synapse in which the active zone (red) and the postsynaptic density (grey) are highlighted. (*b*) Schematic of the active zone, magnified from the grey rectangle (dotted line) in *a*. The active zone is a complex of multidomain proteins that contains ELKS as a central component, and interacts with the presynaptic membrane, synaptic vesicles and the cytoskeleton to mediate synaptic vesicle fusion (adapted from Wang *et al.* [30]).

Extensive research has provided evidence that the active zone is important for the speed and spatial precision of synaptic vesicle fusion [22,24]. The active zone was first defined functionally as the site of exocytosis of synaptic vesicles [25]. Around the same time, it was postulated that the gaps between dense projections, visualized by electron microscopy of presynaptic structures opposed to postsynaptic densities, were the physical correlates of exocytotic sites [26]. Compelling studies of the ultrastructure of the frog neuromuscular junction shed detailed light on the repetitive, organized assembly of docked synaptic vesicles and their proteinaceous connections to the target membrane [27]. Biochemical purifications combined with forward and reverse genetic experiments have since revealed that the active zone is composed of a network of multi-domain proteins [22,28,29] (figure 1). This complex contains six protein families that are relatively restricted in their localization to the active zone: ELKS, RIM (Rab3 interacting molecule), RIM-BP (RIM-binding protein), Munc13 (mammalian Unc13), Liprin-α and Piccolo/Bassoon. In addition, the active zone is connected to many presynaptic components that are important for release, including membrane proteins, lipids, Ca^2+^ channels, the SNARE fusion machinery, synaptic vesicle proteins and the presynaptic cytoskeleton [31–33] (figure 1).

Striking work from many laboratories has demonstrated that individual active zone proteins support important functions in release. Munc13, for example, is an essential vesicle priming protein [34–37] through interactions of a central motif called the MUN domain with SNARE proteins [38–41]. RIM interacts with the Munc13 N-terminal sequences to anchor it to the active zone [42–44] and to activate its priming function [45–47]. Together with RIM-BP, RIM also localizes Ca^2+^ channels close to primed vesicles to accelerate fusion and to enhance release probability [48–53], a function that is in part supported by Bassoon [54].

## 3. Active zone scaffolding is redundant across several protein families

These findings suggested that a fundamental function of the active zone in fusion could be to scaffold essential elements to one another to accelerate fusion and to enhance synaptic strength. Central questions remained unresolved, however, including how and where the active zone is assembled, what holds this protein complex together, and what positions it in opposition to postsynaptic receptors. These questions were difficult to address because no single protein knockout resulted in disassembly of the active zone protein complex at vertebrate synapses. Hence, even the most fundamental aspects of active zone scaffolding could not be studied in neurons, but relied instead on biochemical studies.

This limitation was recently overcome in studies in which simultaneous knockout of either all full-length ELKS and RIM proteins [30], or RIM-binding proteins and RIM proteins [52], led to active zone disassembly. This disruption of scaffolding was accompanied by a near complete loss of docked synaptic vesicles. Importantly, in each compound mutant [30,52] the impairments in scaffolding were stronger than the sum of the phenotypes observed by removing a single protein family [48,49,51,55–57]. These phenotypes revealed that scaffolding of the active zone is a shared function across several active zone proteins, and established that there is strong redundancy for such roles.

Several additional experiments have shown redundancy for specific active zone scaffolding functions. For example, RIM proteins tether presynaptic Ca^2+^ channels [48,58], but this effect requires removing both RIM genes; it cannot be reliably detected in single knockouts [59,60]. Similarly, Ca^2+^ channels themselves are redundant, such that when one channel is removed, its loss is compensated by related channels [61,62]. Finally, the active zone targeting of Ca_V_2.1 channels cannot be impaired by removing a single or a few protein interaction motifs in the C-terminus; impairing Ca_V_2.1 targeting instead required removing many interaction motifs [63]. Hence, scaffolding at the active zone is supported by strong redundancy. Removing a single gene or protein interaction site often has little effect on scaffolding because redundant interactions are sufficient to mediate scaffolding functions.

## 4. ELKS genes and expression pattern

The observation that simultaneous deletion of ELKS and RIM disrupted the active zone established that ELKS serves as an active zone scaffold [30]. As discussed further below, studies of synaptic functions of ELKS have suggested diverse roles for ELKS, and the molecular mechanisms of ELKS function have remained enigmatic. We will here provide an in-depth review of ELKS gene and protein structures, evaluate literature on ELKS protein interactions, and assess whether these interactions could account for scaffolding at the active zone and in other cellular compartments.

ELKS, named for the fact that the protein is rich in the amino acids glutamic acid (E), leucine (L), lysine (K) and serine (S), was first identified as a gene translocated onto RET in papillary thyroid carcinoma [64]. Several different groups later independently described ELKS with three different names, Rab6IP2 (Rab6 interacting protein 2), CAST (Cytomatrix at the active zone Associated STructural protein) or ERC (ELKS/Rab6IP2/CAST) [65–67] (table 1). We here use the ELKS nomenclature, crediting the initial discovery.

**Table 1.** ELKS gene and protein names.

gene name	protein name	promoter/splice isoforms	alternative name	References
*Erc1*	ELKS1	ELKS1αA, ELKS1αB, ELKS1βA, ELKS1βB	CAST2 (=ELKS1αB), RAB6IP2A (=ELKS1αB), RAB6IP2B (=ELKS1αA), ERC1A (=ELKS1αA), ERC1B (=ELKS1αB),	Nakata *et al*. [64,68] Monier *et al*. [65] Wang *et al*. [66] Deguchi-Tawarada *et al*. [69] Liu *et al*. [57]
*Erc2*	ELKS2	ELKS2αA, ELKS2αB, ELKS2βA, ELKS2βB	CAST or CAST1 (=ELKS2αB), ERC2 (=ELKS2αB)	Wang *et al*. [66] Ohtsuka *et al.* [67] Kaeser *et al*. [56]
*elks*	ELKS			Monier *et al*. [65] Deken *et al*. [70]
*brp, bruchpilot*	BRP, Bruchpilot	BRP-190, BRP-170	Nc82	Monier *et al*. [65] Wagh *et al*. [71] Kittel *et al*. [72] Matkovic *et al*. [73]

**Figure 2.**
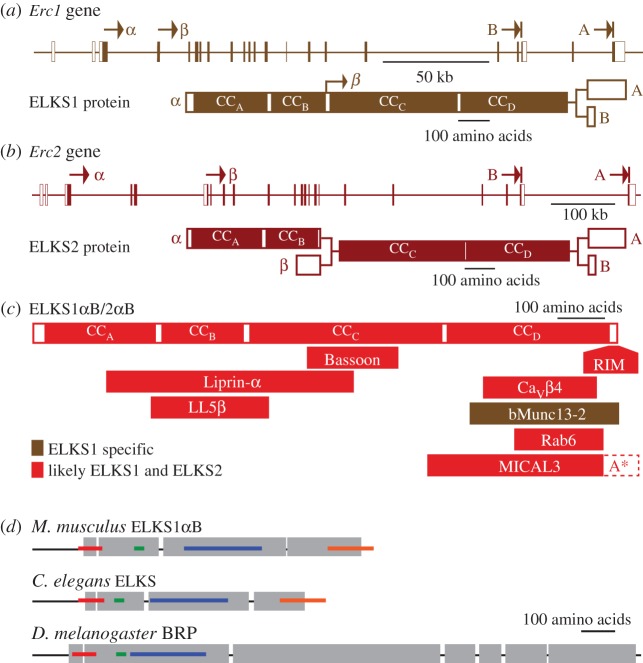
ELKS genes and proteins. (*a*) *Erc1* mouse gene structure (top) and ELKS1 protein structure arising from multiple start codons (α- and β-isoforms) or C-terminal splice variants (A- and B-isoforms). White boxes in the *Erc1* gene indicate untranslated regions (UTR), filled boxes are protein coding regions (modelled after Liu *et al*. [57]). (*b*) *Erc2* mouse gene structure (top) and ELKS2 protein structure arising from alternatively spliced N-terminal exons (α- and β-isoforms, respectively) or C-terminal splice variants (A- and B-isoforms). White boxes in the *Erc2* gene indicate untranslated regions (UTR), shaded boxes are protein coding regions (modelled after Kaeser *et al*. [56]). (*c*) ELKS protein interaction map exemplified by the prototypic αB-isoform. A*, the MICAL3 binding site may extend into the A-variant specific C-terminus. (*d*) Coiled-coil structure prediction (grey boxes) of *Mus musculus* (XP_017176819), *C. elegans* (NP_500329) and *Drosophila melanogaster* (NP_001036535) ELKS1αB/ELKS/BRP using MARCOIL [74]. Coloured bars indicate regions with greater than 25% amino acid sequence identity between species. Red, *M.m.*—^163^REND-RKDE^204^, *C.e.*—^157^REYE-RRDE^198^, *D.m.*—^123^RELG-RKEE^164^; green, *M.m*—^303^NARD-QSKG^320^, *C.e.*—^233^LRER-ESGS^251^, *D.m.*—^247^IARD-QAKG^264^; blue, *M.m*—^436^MKNK-QGDL^668^, *C.e.*—^340^MRMK-QKEL^543^, *D.m.*—^280^MAQK-ESEV^485^; orange, *M.m*—^875^ERRK-GIWA^976^, *C.e.*—^720^ERRQ-GIWA^836^ (modelled after Wagh *et al*. [71]).

Vertebrate genomes express two ELKS genes, *Erc1* and *Erc2*, to produce ELKS1 and ELKS2 proteins (figure 2, table 1). Both genes are large, ranging from approximately 250 to 900 kb of genomic DNA depending on the species and the gene [66,67,69]. Importantly, each gene produces transcript variants that are likely functionally diverse, and the full-length proteins contain approximately 1000 amino acids [56,57,66,68]. At the N-terminus, there is a longer, dominantly expressed α variant that accounts for approximately 95% of ELKS protein in brain for both genes [56,57]. Shorter β variants, which lack the first approximately 350 amino acids and significant protein interaction sites, are only detected at low levels. They arise from a secondary start codon in ELKS1 [57] or an alternative exon 1″ in ELKS2 [56]. The C-termini of both proteins also exist as longer A and shorter B variants due to alternative splicing [56,66]. The B-variant contains a PDZ domain interaction site and is the dominant ELKS variant in neurons. In total, each gene has four transcript variants: αA, αB, βA and βB, of which αB predominates in brain [57]. There are also numerous internal exons that are potentially alternatively spliced in both genes [66,68], but to what extent these transcripts lead to functionally relevant variation in ELKS proteins is not known.

ELKS, like most active zone proteins, is conserved throughout species. Humans express two *ERC* genes, each of which produces protein with approximately 99% sequence identity with the corresponding mouse homologue [66]. *Caenorhabditis elegans* contains a single homologous gene called *elks* [65,70]. *Drosophila* expresses a partial homologue, *bruchpilot* (*brp*), which shares sequence similarity with ELKS in its N-terminal half, including alternative start sites [65,71,73], but has an unrelated C-terminal portion (figure 2*d*). The ELKS protein consists of coiled-coil sequences (grey boxes in figure 2*d*) that are mostly conserved across species with several long stretches of greater than 25% amino acid sequence identity (coloured bars in figure 2*d*). At the C-terminus *Drosophila* BRP diverges, lacking sequences for several important ELKS protein interactions (figure 2*c*), and it shows instead similarity to plectin and myosin heavy chains [65,71]. This is important when comparing synaptic functions of ELKS with BRP, because several important functions of ELKS or BRP have been mapped to their C-terminal sequences [72,75–79].

ELKS proteins are enriched in the nervous system, where they are expressed primarily as the ELKS1αB and ELKS2αB isoforms [57,66,67]. The longer C-terminal A variant of ELKS1 appears to be mostly expressed in peripheral tissues [57,66], whereas expression of ELKS2A has not been well characterized. Within the nervous system, ELKS1 and ELKS2 proteins are synaptic proteins as assessed by confocal microscopy. Immunogold electron microscopy with an antibody that likely recognizes ELKS1 and ELKS2 indicates active zone localization at the ultrastructural level [67], consistent with ELKS interactions with several active zone proteins [80,81]. There is, however, evidence to support that some ELKS is localized outside of active zones, for example in the Golgi apparatus [65] and at the cellular cortex associated with microtubules [82].

## 5. ELKS protein structure and interactions

While ELKS has no clear domain structure beyond the presence of stretches of α-helical coiled-coil regions (figure 2*d*) [65–67], assessment of sequence homology among various ELKS proteins has led to the definition of four coiled-coil regions termed CC_A_–CC_D_ that are conserved between isoforms (figure 2*a,b*) [83]. In ELKS B isoforms, the CC_A_–CC_D_ domains are followed by a PDZ domain-binding motif (amino acids …GIWA). Affinity purifications have identified a multitude of protein–protein interactions between ELKS and proteins within and outside of the synapse (figure 2*c*). The C-termini of ELKS1αB/2αB bind to the PDZ domain of RIM with relatively high affinity [66,67,84], and to the PDZ domain of the syntenin [85]. In addition, both ELKS isoforms bind to presynaptic Liprin-α proteins [80] via the N-terminal coiled-coil regions (CC_A_–CC_C_), and the gene structure suggests that these interactions are specific to α-ELKS isoforms [56,57]. The C-terminal CC_D_ also interacts with β subunits of the voltage-gated Ca^2+^ channel (Ca_V_β), in particular Ca_V_β4 [86–88], and the central coiled-coils (CC_B_ and CC_C_) bind to the active zone scaffolds Bassoon and Piccolo [79]. Finally, the N-terminal region of bMunc13-2, but not of Munc13-1, has a specific interaction with the C-terminus of ELKS1 [78], but not of ELKS2.

ELKS1 interactions have also been extensively studied in the context of cortical microtubule stabilizing complexes (CMSCs). ELKS1 binds to and colocalizes with two components of CMSCs: LL5β, a PIP_3_-binding protein (CC_A_–CC_C_), and MICAL3 (CC_D_), a monoxygenase involved in cytoskeletal remodelling [82,89,90]. In addition, ELKS1 and ELKS2 bind with their CC_D_ region to Rab6 [65,91], a small GTPase that may be targeted to CMSCs in addition to its prominent localization in the in Golgi [65,89,92,93].

## 6. ELKS knockout phenotypes: redundancy to support active zone structure

The coiled-coil structure and the numerous protein interactions of ELKS led to the hypothesis that ELKS acts as an essential scaffold to support active zone structure [66,67,79,94]. However, initial knockout studies in mammals and invertebrates indicated a more complex situation. In *C. elegans*, ELKS null mutants exhibit no deficits in the localization of the active zone proteins RIM or UNC-13, the Munc-13 homologue, to synapses in the dorsal nerve chord [70]. Similarly, ELKS was not required for the clustering of synaptic vesicles to the dorsal chord of HSN synapses [95]. In mice, conditional knockout of the α-isoforms of ELKS1 and ELKS2 in cultured hippocampal neurons, removing approximately 95% of all ELKS, did not strongly impair synapse ultrastructure or localization of other active zone proteins [57,83]. Knockout of only ELKS2α in mice, however, showed a small decrease in the number of vesicles per bouton and a slight increase in the solubility of RIM in a biochemical fractionation, accompanied by an unexpected increase in inhibitory synaptic strength [56]. A separately generated ELKS2α knockout mouse showed a decrease in the size of the presynaptic ribbon of rod photoreceptor synapses, paralleled by a significant increase in the expression of ELKS1 [96]. These studies established that removal of α-ELKS isoforms does not lead to disassembly of active zones or synapses, arguing against major autonomous scaffolding roles for α-ELKS. It remains possible that when β-ELKS isoforms are removed in addition to α-isoforms, scaffolding roles that are non-redundant with other protein families may be uncovered.

**Figure 3.**
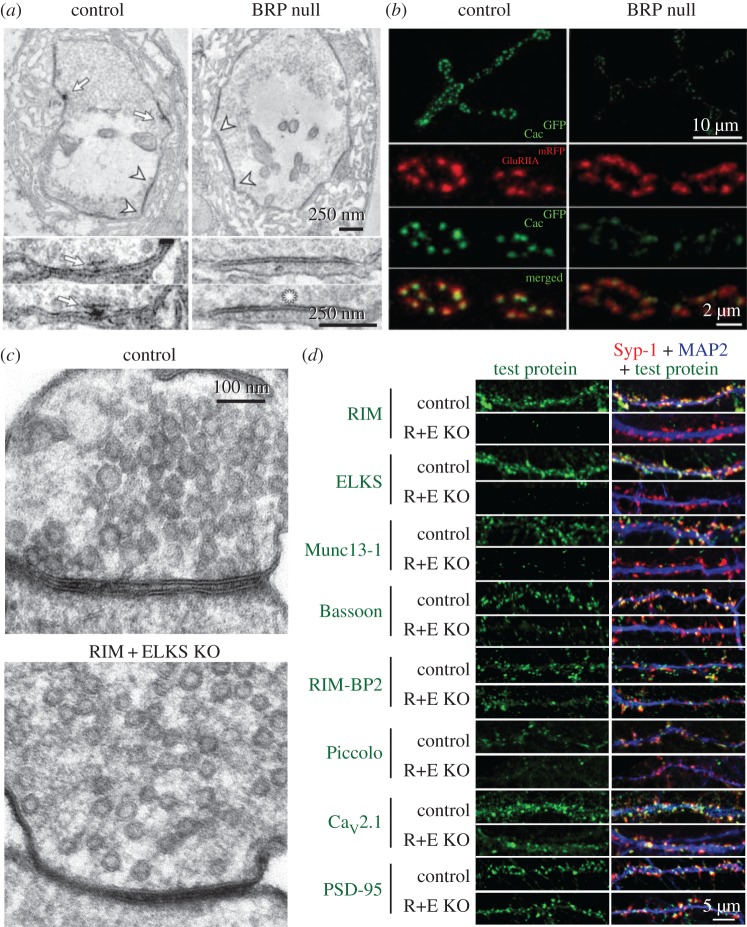
ELKS as a presynaptic scaffold. (*a,b*) Removal of partial ELKS homologue BRP leads to disruption of the presynaptic T-bar (*a*) and the reduced recruitment of presynaptic Ca^2+^ channels (*b*, Cac^GFP^) at the fly NMJ (adapted from Kittel *et al.* [72]). (*c,d*) Simultaneous deletion of RIM and ELKS (R + E KO) leads to loss of vesicle docking (*c*) and disruption of the active zone scaffold (*d*) in hippocampal neurons. Syp-1 (synaptophysin-1) is a synaptic vesicle marker used to label synaptic terminals (adapted from Wang *et al.* [30]).

*Drosophila* BRP, however, is an essential scaffold. The T-bar of the *Drosophila* neuromuscular junction (NMJ), an electron-dense structure that forms an umbrella-shaped density at the presynaptic membrane of the fly NMJ, is almost completely disrupted in BRP null mutants (figure 3*a,b*) or upon BRP knockdown [71,72]. BRP, therefore, appears more essential to presynaptic structure than vertebrate ELKS, or any vertebrate active zone protein tested so far, perhaps reflecting that the T-bar is a specialized structure not present at vertebrate active zones. There is, however, striking similarity with the synaptic ribbon, an electron-dense assembly at release sites of specialized cell types, including retinal photoreceptors, bipolar cells, and hair cells in the inner ear. There, the integrity of the electron dense structure also strongly depends on the presence of a single protein, Ribeye [97,98].

The first vertebrate genetic evidence in support of a strong scaffolding role for ELKS was provided by simultaneous knockout of ELKS1α/2α together with RIM1/2 (figure 3*c,d*). Simultaneous deletion of RIM and ELKS resulted in a strong disruption of the active zone with loss of Munc13-1, Bassoon, Piccolo and RIM-BP2, but not of Liprin-α [29,30]. Importantly, such strong scaffolding roles were not observed in individual knockouts for either RIM or ELKS, apart from reductions in Munc13 [43,46,48] or increased solubility of RIM [56] in RIM or ELKS knockouts, respectively. In addition, synaptic vesicle docking and tethering was very strongly impaired upon simultaneous deletion of RIM and ELKS, further supporting a profound structural disruption of the active zone in these mutants. Hence, while ELKS plays a structural role, its loss can be compensated for by redundant protein interactions between other active zone components. These data, along with the mild structural effects seen in knockouts of RIM or ELKS alone, illustrate a hallmark feature of the active zone: it is an extensively interconnected protein network that is remarkably resilient to genetic removal of individual components. Only once redundancy is removed can the structure be significantly disturbed.

## 7. ELKS knockout phenotypes: diverse active zone functions

**Figure 4.**
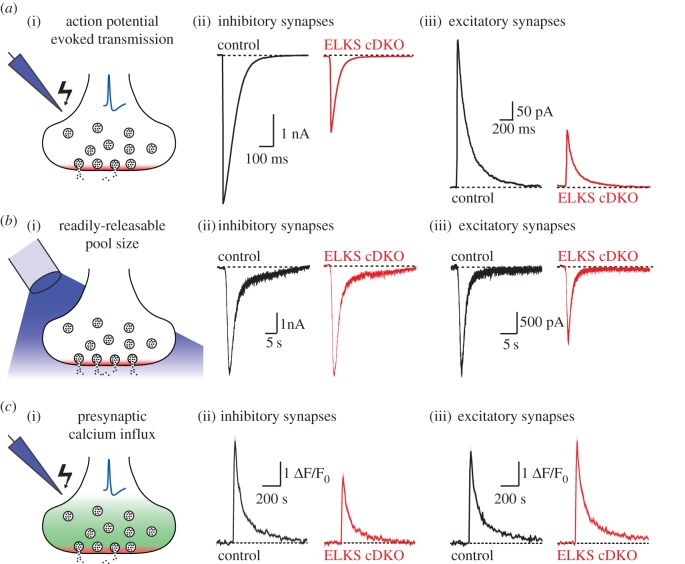
ELKS knockout phenotypes in hippocampal neurons. (*a*) Action potential-evoked release (i) is decreased at inhibitory (ii) and excitatory (iii) hippocampal synapses, exemplified by postsynaptic current recordings (adapted from Held *et al.* [83]). (*b*) The RRP as assayed by the application of hypertonic sucrose (i) is impaired at excitatory (iii) but not inhibitory (ii) hippocampal synapses, illustrated by postsynaptic current recordings (adapted from Held *et al.* [83]). (*c*) Presynaptic Ca^2+^ influx, measured in response to a single action potential via Fluo5F fluorescence, is impaired at inhibitory (ii) but not excitatory synapses (iii, adapted from Liu *et al.* [57] and Held *et al.* [83]).

Next to studying structural roles at the active zone for ELKS, several studies have assessed defects of synaptic transmission in ELKS mutants. In knockout mice, ELKS removal consistently has an effect on neurotransmitter release, with the exception of striatal dopamine secretion [99], but the exact phenotypes and synaptic mechanisms vary significantly between synapses and alleles. In cultured hippocampal neurons, simultaneous knockout of ELKS1α and ELKS2α results in an approximately 50% reduction in action potential evoked release at inhibitory and excitatory synapses (figure 4). The synaptic mechanisms behind these release deficits are distinct, however. At inhibitory synapses, ELKS1α/2α knockout leads to decreased presynaptic Ca^2+^ influx, resulting in a lower synaptic vesicle release probability, without changing the size of the RRP [57]. Conversely, ELKS1α/2α knockout induces a decrease in RRP size but no change in presynaptic Ca^2+^ influx at excitatory synapses [83].

Knockout of single ELKS isoforms has further supported diverse ELKS functions at synapses. ELKS2α knockout leads to increased evoked inhibitory postsynaptic current amplitudes in cultured hippocampal neurons and in acute hippocampal slices [56]. This increase was specific to inhibitory neurons, while excitatory synapses were either not [56] or only mildly affected [91] by knockout of ELKS2α. Finally, ELKS2α removal also decreased potentials in electroretinograms, suggesting impairments in synaptic transmission consistent with the effects on ribbon structure in rod photoreceptors in these mice [96].

Invertebrate studies also reveal variable synaptic phenotypes. Removal of ELKS in *C. elegans* does not have a significant effect on evoked excitatory postsynaptic currents at the cholinergic NMJ [70]. *Drosophila* BRP mutants, in keeping with their disruption of the T-bar, have an approximately 60% reduction in evoked excitatory junction currents and a decrease in vesicular release probability [72]. This is likely caused by a mislocalization of presynaptic Ca^2+^ channels (figure 3*b*). However, truncated BRP mutants that lack only the C-terminal 522 amino acids partially restore the presence of T-bars and largely restore localization of Ca^2+^ channels, but notably fail to rescue eEJC amplitudes [76,77], indicating that mechanisms other than Ca^2+^ channel localization contribute to the BRP knockout phenotype. Remarkably, truncation of the last 17 amino acids of BRP (*brp*^nude^) restores T-bar morphology and single evoked synaptic vesicle release, but does not restore vesicle tethering at the T-bar, short-term plasticity or the survival and locomotion deficits of BRP mutants [76]. While the survival deficits in *brp*^nude^ mutants may be due to synaptic transmission deficits, isolated changes in short-term synaptic plasticity likely do not cause lethality [100,101]. Hence, BRP, like ELKS, may have additional cellular functions that go beyond mediating exocytosis.

## 8. ELKS functions outside of the active zone

ELKS1 also has roles in membrane trafficking in non-neuronal cells. ELKS is localized to CMSCs via its interaction with LL5β at the cell cortex of HeLa cells [82]. There, through direct interactions with Rab6 and indirect interactions with Rab8A via MICAL3, ELKS is important for targeted exocytosis of vesicles containing Rab6A and Rab8A GTPases [89,90]. In ELKS knockdown cells, Rab6/8A vesicles accumulate at the cell cortex at CMSCs [90]. Exocytotic events still occur at these sites, though they are temporally delayed, indicating that ELKS may be important for efficient capture and docking of vesicles at the fusion site, but not for fusion itself. ELKS knockdown only has small effects on the accumulation of CMSC proteins such as CLASPs and LL5β [82], suggesting that it is not an essential autonomous scaffold for these proteins. The CMSC complex is also present at the leading edge of migrating cells where targeted exocytosis can facilitate the turnover of focal adhesions [102]. Knockdown of ELKS in these cells interferes with this process, causing a reduction in focal adhesion turnover and a decrease in invasive behaviours in cancer cell lines [103,104]. It is important to note that roles of non-active zone localized ELKS could contribute to electrophysiological phenotypes described in ELKS loss-of-function experiments in neurons.

## 9. Molecular mechanisms of ELKS

**Figure 5.**
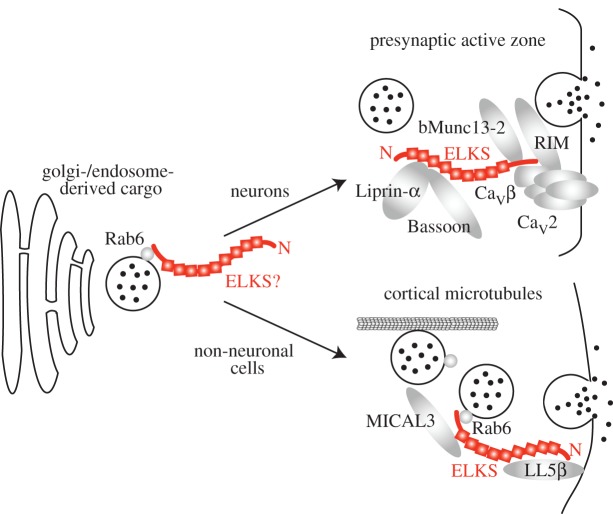
Current working model of ELKS functions. ELKS functions at various steps along the secretory pathway. ELKS may be involved in cargo assembly in the Golgi apparatus or in an endosomal compartment. In neurons, the majority of ELKS is localized to the presynaptic nerve terminal, where it interacts with multiple active zone proteins to support neurotransmitter release. In non-neuronal cells, ELKS was identified in a complex that anchors cortical microtubules to the plasma membrane and supports the capture and exocytosis of vesicular cargo.

While the functional roles of ELKS are diverse, we propose that a unifying feature of ELKS mechanisms is a scaffolding role. This role, likely executed in several steps of the secretory pathway (figure 5), can mediate the tethering of other proteins, the capturing of vesicular cargo or the membrane attachment of cytoskeletal elements through interactions with membrane-associated proteins. Given the interconnected nature of the active zone and the other scaffolding complexes ELKS participates in, a single set of interactions that is engaged uniformly across synapses and cellular compartments is unlikely to account for ELKS knockout phenotypes. The diverse functions are likely mediated by engaging different molecular interactions depending on the protein nano-environment. Hence, it is necessary to consider individual ELKS interaction partners and their differential localization to account for the roles of ELKS as revealed in loss-of-function phenotypes.

### 9.1. Ca^2+^ channel subunit interactions to boost Ca^2+^ influx

In ELKS1α/2α knockouts, Ca^2+^ influx deficits were restricted to inhibitory synapses and were not accompanied by reductions in Ca^2+^ channel expression or by a loss of their synaptic localization [57]. These data suggest that loss of ELKS reduces calcium influx at inhibitory synapses during an action potential by reducing the time window or voltage range during which the channels are open. ELKS has been shown to bind to Ca_V_βs, most prominently Ca_V_β4, in affinity purifications [86–88]. Ca_V_β subunits are auxiliary subunits of the Ca^2+^ channels that promote their surface expression and change channel kinetics [105]. In transfected, heterologous cells, coexpressing ELKS2 with Ca^2+^ channels, including Ca_V_β4, shifted the voltage-dependent activation of the channel, allowing it to open at more hyperpolarized voltages [86]. Hence, loss of such an interaction at synapses might reduce the amount of Ca^2+^ influx during an action potential, because channels would be open for a more narrow time window. It is, therefore, possible that ELKS–Ca_V_β interactions account for reduced Ca^2+^ influx at inhibitory synapses. Since mammals express four different genes for Ca_V_β, ELKS–Ca_V_β4 interactions could be engaged in a synapse-specific manner. For example, restricted localization of Ca_V_β4 to inhibitory synapses could account for a phenotype specific to these synapses. However, it is currently not known whether Ca_V_β subunits are expressed and localized in a synapse-specific manner at synapses where ELKS functions have been evaluated. Future experiments should address this point and should determine whether synapses that express specific Ca_V_β subunits [106] have Ca^2+^ influx deficits in ELKS mutants.

### 9.2. Munc13 and other interactions to modulate vesicle priming

Munc13s are encoded by three genes in vertebrates and are essential for generating the RRP [34,35,107,108]. Munc13-1 is the primary isoform in hippocampal cultures, whereas bMunc13-2, the brain-specific variant of Munc13-2, is present in only a small subset of synapses [35,78,109]. The N-terminus of bMunc13-2 binds specifically to the ELKS1 CC_D_, and this interaction is important for the recruitment of bMunc13-2 to synapses [78]. Furthermore, rescue of Munc13-1/2 knockout neurons with bMunc13-2 constructs missing the ELKS1 binding region showed reduced RRP size compared to full-length bMunc13-2 rescue. ELKS1–bMunc13-2 interactions are, therefore, likely to control RRP size through bMunc13-2. However, in the hippocampus, this priming activity is not widespread because most synapses do not express bMunc13-2 [78,108]. Hence, this interaction provides for a mechanism to boost RRP specifically at synapses that co-express bMunc13-2 and ELKS1 [78]. This mechanism could also potentially explain the increased RRP in ELKS2α knockout mice [56], where deletion of ELKS2α would result in boosting of the RRP through an enhanced role of ELKS1–bMunc13-2-mediated vesicle priming.

Importantly, Munc13-2 knockout mice do not have an RRP deficit in cultured neurons [35], but ELKS knockout mice do, and rescue of this deficit does not require the bMunc13-2 binding sequences of ELKS [83]. Hence, the bMunc13-2 interaction with ELKS1 does not fully explain the impairment of RRP in ELKS knockouts, and additional priming activities must be present in ELKS. Rescue experiments in ELKS1α/2α knockouts revealed that the N-terminal coiled-coil domains of ELKS1 (CC_A_–CC_C_) were required to rescue the RRP deficit [83]. The required domains correspond to known interaction sequences for two active zone proteins, Liprin-α and Bassoon [79,80]. While either or both proteins may be involved in modulating RRP, Liprin-α is the more promising candidate because ELKS1 rescue constructs which contain the entirety of the Bassoon binding sequence failed to rescue RRP size. Alternatively, unknown interactions within CC_A_–CC_C_ could contribute to vesicle priming.

### 9.3. ELKS–RIM interactions

The best characterized ELKS binding partner, RIM, is known to control RRP size as well as presynaptic Ca^2+^ influx [43,46,48,58,110–112]. While this may suggest that ELKS phenotypes may be explained by loss of RIM recruitment to the active zone, current data do not support this model. First, the reduction in Ca^2+^ influx at inhibitory synapses in the ELKS1α/2α knockouts was not accompanied by a significant loss of RIM at inhibitory synapses [83], nor by a loss of Ca^2+^ channel synaptic localization, a phenotype found in RIM knockouts [48,57,58]. Second, at excitatory synapses the reduction in RRP size in ELKS1α/2α knockouts could be rescued by ELKS fragments that do not include the RIM-binding sequence [83], establishing that this effect is not mediated by ELKS C-terminal interactions with RIM PDZ domains. While these data do not exclude a functional role for ELKS–RIM interactions, they suggest that these interactions are not the basis for the release deficits observed in the ELKS knockouts.

### 9.4. ELKS–Rab6 interactions to support cargo assembly and trafficking

A potential role for ELKS in neurons involves ELKS–Rab6 interactions at the Golgi. When ELKS was identified as a Rab6 binding partner it was proposed to be involved in trafficking from the endosome to the trans-Golgi network [65]. Rab6 proteins have been implicated in intra-Golgi transport, retrograde Golgi–ER transport and targeted exocytosis at the cell cortex [89,93,113–115]. ELKS has also been shown to be trafficked from the Golgi in distinct active zone precursor vesicles that contain Bassoon and Piccolo [116]. Hence, interactions between ELKS and Rab6 at the Golgi could be involved in the trafficking of active zone or other synaptic components, thereby controlling synaptic function. Such functions could be reflected in defects that go beyond exocytosis, for example in membrane trafficking that involves the presynaptic endosome.

An alternative model is that ELKS interacts with Rab6 in the cell periphery, for example at CMSCs, which are involved in targeted exocytosis [117]. There, ELKS participates in a complex that contains LL5β and MICAL3, proteins that are not known to be present at the active zone. The release of Rab6/Rab8 positive cargo is impaired upon knockdown of ELKS [82,89,90]. Remarkably, the Rab6 binding site and the N-terminal sequences of ELKS are required for rescue of this vesicle accumulation phenotype [89], establishing a scaffolding role that requires several protein interaction domains.

## 10. Conclusion and outlook

Though ELKS proteins remain enigmatic components of the active zone, determining their exact structural and functional role at the active zone will be crucial to our understanding of synaptic transmission. Human genetic studies further indicate that ELKS may play a role in autism spectrum disorders and developmental speech deficits [118,119], suggesting that developing a deep knowledge of ELKS is important for understanding neurological disease. Recent work has established that ELKS is a redundant scaffold at the active zone, and that disruption of this scaffold impairs fusion of synaptic vesicles. Parallel studies in non-neuronal cells have shown scaffolding and exocytotic functions for constitutive secretion of other cargo. This leads to a working model in which ELKS localizes to exocytotic hot spots, where it is likely involved in the scaffolding of other proteins to establish a secretory site and in the capture and release of the exocytotic carrier (figure 5). The exact mechanisms and phenotypes depend on the protein nano-environment at the secretory site.

While we have mostly discussed mechanisms of individual ELKS domains, scaffolding requires the simultaneous engagement of multiple interactions, and future studies will need to address this point. In the case of ELKS, it will be necessary to determine which interactions target it to the secretory site, and which interactions are downstream for the recruitment of cargo or other proteins. We are entering a new stage in our understanding of the structural composition and molecular function of the active zone, in part thanks to the efforts to understand the scaffolding mechanisms that are mediated by ELKS. Future work should emphasize looking at the structure of this protein complex *in situ* at sub-nanometre resolution, expanding on a few available studies that started this fascinating enterprise [120,121]. This will shed further light into the striking nanostructure that underlies the precision of transcellular signalling at a synapse [23].

## Data accessibility

This article has no additional data.
